# Targeted next generation sequencing identifies somatic mutations and gene fusions in papillary thyroid carcinoma

**DOI:** 10.18632/oncotarget.17412

**Published:** 2017-04-25

**Authors:** Zheming Lu, Yujie Zhang, Dongdong Feng, Jindong Sheng, Wenjun Yang, Baoguo Liu

**Affiliations:** ^1^ Key Laboratory of Carcinogenesis and Translational Research (Ministry of Education), Laboratory of Biochemistry and Molecular Biology, Beijing, China; ^2^ Key Laboratory of Carcinogenesis and Translational Research (Ministry of Education), Department of Head and Neck Surgery, Peking University Cancer Hospital & Institute, Beijing, China; ^3^ Key Laboratory of Fertility Preservation and Maintenance (Ministry of Education), Cancer Institute of the General Hospital, Ningxia Medical University, Yinchuan, China

**Keywords:** papillary thyroid carcinoma (PTC), cancer panel, fusion gene, somatic mutation

## Abstract

138 papillary thyroid carcinoma (PTC) samples were assessed for somatic mutation profile and fusion genes by targeted resequencing using a cancer panel (ThyGenCap^TM^) targeting 244 cancer-related genes and 20 potential fusion genes. At least one genetic alteration (including mutations and fusion genes) was observed in 118/138 (85.5%) samples. The most frequently mutated gene was *BRAF* V600E (57.2%). Moreover, we identified 11 fusion genes including eight previously reported ones and three novel fusion genes, *UEVLD-RET*, *OSBPL9-BRAF*, and *SQSTM1-NTRK3*. Alterations affecting the mitogen-activated protein kinase (MAPK) signaling pathway components were seen in 69.6% of the PTC cases and all of these driver mutations were mutually exclusive. Univariate analysis ascertained that the fusion genes were strongly associated with distinct clinicopathological characteristics, such as young age, local invasion, extensive metastasis, and disease stage. In conclusion, our approach facilitated simultaneous high-throughput detection of gene fusions and somatic mutations in PTC samples.

## INTRODUCTION

Papillary thyroid carcinoma (PTC) is the most common thyroid malignancy in adults, the incidence of which has been soaring in recent decades [1–3]. Over the past 20 years, new discoveries in the pathogenesis of PTC have given insight into better diagnostic, prognostic, and therapeutic procedures for patients with thyroid cancer [2, 4, 5]. Previous genetic studies have reported point mutations of the *BRAF* and the *RAS* genes, as well as fusions involving the RET and NTRK1 tyrosine kinases [6]. More recently, Integrated Genomic Characterization of PTC by the Cancer Genome Atlas (TCGA) project reduced the proportion of PTC cases with unknown oncogenic driver from 25% to 3.5% [7]. Nearly all of these driver mutations are mutually exclusive thus accounting for the classification of PTC in several genetically defined subgroups. Therefore,understanding genetic lesions is necessary for elucidating distinct clinicopathological characteristics,such as clonal evolution, risk stratification, and therapeutic targets.

With the advent of next-generation sequencing (NGS), a more complete biological characterization of a tumor can be attained at the molecular level by allowing simultaneous analysis of large regions of the genome, and offering high-sensitivity detection of mutations, and quantitative assessment of mutant alleles [8, 9]. Recently, molecular genetics of PTC have been studied on a different scale including whole-genome sequencing, whole-exome sequencing, and whole-transcriptome sequencing [7, 9]. These methodologies are complementary to each other and are essential for genetic discovery projects. Further, targeted sequencing of multiple specific genomic regions may offer an easier and less expensive alternative strategy in routine molecular diagnostics of cancer allowing a more detailed cancer genetic lesions to be obtained [10, 11].

In the present study, we developed a targeted, massively paralleled sequencing assay for 244 cancer-related genes and 20 fusion genes by a homebred Thyroid Cancer Panel (ThyGenCap^TM^ panel),designed as a comprehensive diagnostic test for mutations and gene rearrangements of PTC in an efficient and cost-effective manner. Our assay has allowed improvements on earlier approaches, most importantly by expanding the spectrum of mutations and including complex genomic rearrangements that are potentially detectable by the assay [9, 12].

## RESULTS

### Quality assessment of the targeted sequencing data

Patient characteristics are described in Supplementary Table 1. Paired specimens of PTC and matched blood samples were used for analysis. The mean value of raw variant coverage was 18489435 reads for each sample, and the variant coverage ranged from 1354085 to 84293424 reads. On average 97.58% of all reads could be mapped back to the genome and 60.48% of all reads were mapped to our designed target regions (43.89%-73.89%). This indicated a high capture efficiency of the probes. The mean read length was 150 base pairs and the mean coverage of the 138 samples analyzed was 288×(124×-928×).

### Somatic mutations in PTC

**Figure 1:**
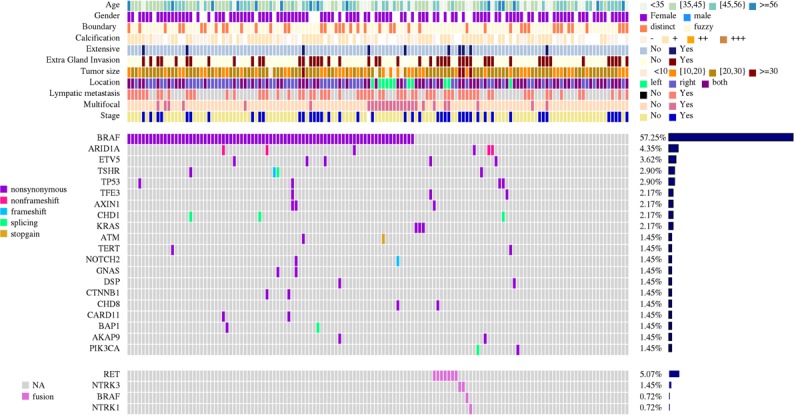
Detailed description of mutation and gene rearrangement landscape of 138 cases of papillary thyroid carcinoma Genes mutated in two or more cases are listed vertically in decreasing order of prevalence. Colored rectangles indicate mutation categories observed in a given gene and tumor. Results of gene rearrangement analysis are shown in the bottom panel. Patient demographics as well as tumor classifications and molecular features, such as age, gender, boundary, calcification, extensive metastasis, extragland invasion, and lymphatic metastasis, are indicated in the boxes on the right.

The number of mutated genes per tumor was 1.39 (range 0–4). Overall, 203 somatic mutations including 181 SNVs (1 splicing, 3 stopgain, and 177 nonsynonymous), 17 deletions, and 4 insertions in 77 genes were identified in the PTC samples. Somatic mutations with mutant allele frequency greater than 10% were all confirmed by Sanger sequencing. Point mutations were most frequently observed in *BRAF* (80 mutations in 138 patients,58.0%), *BRAF* V600E mutation accounted for all the affected cases except for one case that showed *BRAF* V601K. The mutational frequency of other mutations was lower than 5%. A list of the genes that occurred in at least 2 PTC patients is shown in Figure 1. The majority of the tumors (95/138, 68.8%) harbored at least one driver-gene mutation (Supplementary Table 3), 57.2% (79/138) showed concurrent mutations in two or more genes (Figure 1 and Supplementary Table 3), and 24 (18.16%) tumors showed no somatic mutation in the 244 genes analyzed.

### Detection and validation of fusion genes

**Figure 2:**
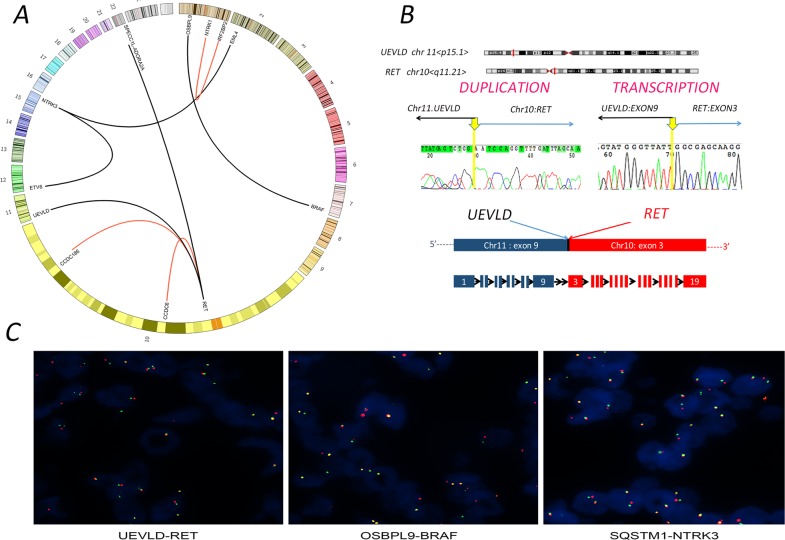
Gene fusions identified from papillary thyroid carcinoma (PTC) cases (**A**) Circos plot of eleven fusion genes in tracks with chromosomes circularly arranged. The corresponding genes are labeled inside the circus. Red lines represent novel fusion genes, and black lines represent already reported ones. (**B**) Validation of novel candidate fusion genes *UEVLD-RET* by fusion-specific DNA and RNA sequencing. Chromosomal ideograms show *UEVLD-RET* gene fusions between *UEVLD* (chr.11 p15.1) and *RET* (chr.10 q11.21). Sanger sequencing chromatograms show breakpoint regions on gene-level with exact single-base resolution fusion point indicated (Hg19 coordinates) and confirmation of the in-frame fusion of *UEVLD* (exon 9) and *RET* (exon 3) by fusion-specific transcription. The arrowheads indicate *RET* fusion points between *UEVLD* and *RET* gene sequences and the genomic coordinates of the RNA fusion junction localized at Chr11:18541431/Chr1043101044. Each gene in the fusion plot is drawn 5′ to 3′. The breakpoints of *RET* are exons 3, which allow the fusion to harbor the kinase domain of RET. Predicted chimeric protein structure of rearrangements inferred from genomic and transcription data are displayed under electropherograms. (**C**) Three novel fusions, *UEVLD-RET*, *OSBPL9-BRAF*, and *SQSTM1-NTRK3*, validated by break-apart fluorescence *in situ* hybridization (FISH) assay. FISH reveals separations of the 5′ probe (red) from the 3′ probe (green) signals. The yellow signal indicating of a rearrangement in tumor cells.

We identified both known and novel fusions, i.e. new partners of previously described fusions, in 11 (7.97 %) of the 138 informative cases (Table 1 and Figure 2). Among them, *UEVLD-RET*, *OSBPL9-BRAF* and *SQSTM1-NTRK3* were three novel fusion genes. *RET* fusions were the most frequent (7/138, 4.35%), including three previously reported partners and one novel unique *RET* fusions (Table 1 and Figure 2). Sequencing analysis of the PCR products confirmed the presence of fused genes on DNA and transcription level in the corresponding tumors; an example for *UEVLD-RET* fusion gene is shown in Figure 2B. Finally, the presence of the fusion between these two genes was confirmed by two-color FISH using probes corresponding to 5′ and 3′ of the breakpoints. Figure 2C showed the representative validation by FISH involving threenovel candidate fusion genes *UEVLD-RET*, *OSBPL9-BRAF* and *SQSTM1-NTRK3*.

**Table 1:** Fusion genes identified from 138 PTC

PatientID	Gender	Age	Gene fusiondetection	Novel ornot	DNAvalidation	RNAvalidation	FISHvalidation	Reference
14	Female	47	SPECC1L-RET	No	Yes	Yes	Yes	25
76	Male	30	UEVLD-RET	Yes	Yes	Yes	Yes	NEW
94	Female	51	CCDC6-RET	No	Yes	Yes	Yes	20
130	Male	40	CCDC6-RET	No	Yes	Yes	Yes	20
98	Female	52	CCDC6-RET	No	Yes	Yes	Yes	20
125	Male	40	CCDC6-RET	No	Yes	Yes	Yes	20
124	Female	29	CCDC186-RET	No	Yes	Yes	Yes	23
138	Male	19	IRF2BP2-NTRK1	No	Yes	ND	Yes	22
135	Female	37	SQSTM1-NTRK3	Yes	Yes	ND	Yes	NEW
133	Female	44	EML4-NTRK3	No	Yes	ND	Yes	21
89	Male	25	OSBPL9-BRAF	Yes	Yes	Yes	Yes	NEW

### Correlation between MAPK pathway, *BRAF*, and fusion genes and clinicalpathological characteristics

**Figure 3:**
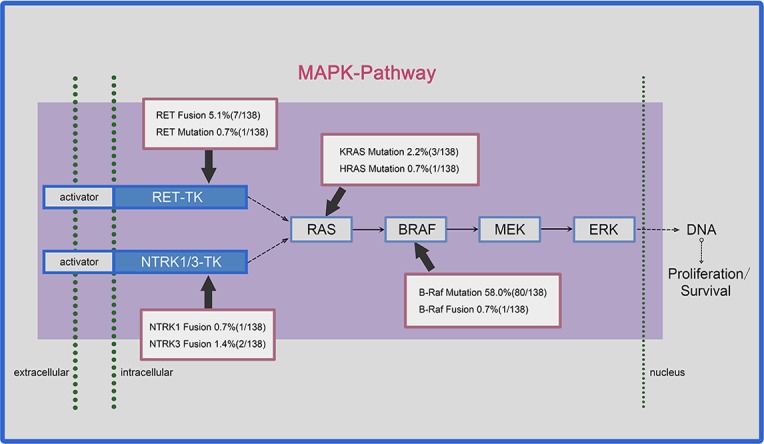
Prevalence of somatic mutations and chromosomal rearrangements in papillary thyroid carcinoma (PTC) affecting the MAPK pathway The MAPK pathway is driven by activating mutations, including *RET*, *BRAF* and *RAS* mutations; and gene arrangements, including the *RET* gene, the *NTRK1/3* gene, and the *BRAF* gene. Both ways of alterations constitutively activate the downstream components of the MAPK pathway, resulting in multiple responses at the transcriptional level and leading to the expression of factors promoting cell proliferation and survival.

We observed that 69.6% of the PTC cases had genetic alterations leading to constitutive activation of the MAPK pathway, including point mutations of *BRAF* (80, 58.0%),*KRAS* genes and *HRAS* genes (4, 2.9%), *RET* genes (1, 0.7%), as well as gene fusions involving the *RET* (7, 5.1%), *BRAF* (1, 0.7%), and *NTRK1* and *NTRK3* tyrosine kinases (3, 2.2%) (Figure 3). These mutations were almost mutually exclusive, suggesting that activation of a single effector of this pathway is sufficient for cell transformation. We further analyzed the correlation between MAPK pathway alteration and the clinicopathological features of PTC. As shown in Table 2, MAPK pathway alteration was significantly associated with advanced tumor stage III/IV (*P*<0.05). Interestingly, the fusion genes were strongly associated with distinct clinicopathological characteristics, including younger age at diagnosis (*P*=0.013), extensive metastasis (*P*=0.025), local invasion (*P*=0.048), large tumor size (*P*=0.003), and disease stage (*P*=0.035) (Table 2).

**Table 2:** MAPK pathway, BRAF and fusion genes analysis with clinicopathology indexes

Variables	MAPK pathwaya			Fusion genesc			BRAF mutationd		P valuesb
	YES	NO	P values^b^	YES	NO	P values^b^	YES	NO	
Total	96	42		11	127		80	58	
Gender			0.393			0.139			0.844
Male	26	8		5	29		19	15	
Female	70	34		6	98		61	43	
Age(years)			0.872			0.013			0.055
≤30	9	5		4	10		5	9	
>30	86	38		7	117		75	49	
Lymphatic metastasis			0.137			0.112			0.733
Metastasis	49	15		10	54		37	27	
Non-metastasis	47	27		1	73		43	31	
**Extensive metastasis^e^**			0.276			0.025			0.648
Non-extensive	88	41		8	121		75	54	
Extensive	8	1		3	6		5	4	
**Extraglandular invasion^f^**			0.177			0.048			0.870
Non-invasion	58	31		4	86		54	36	
Invasion	37	11		7	41		26	22	
**Border^g^**			0.842			0.172			0.513
Clearance	28	14		1	42		27	16	
Obscure	67	28		10	85		53	42	
Tumor size(mm)			0.258			0.003			0.673
≤10	35	22		0	55		32	23	
>10	61	20		11	72		48	35	
**Calcification^g^**			0.2			0.71			0.181
Non-calcification	16	15		3	28		14	17	
Calcification	79	28		8	99		66	41	
**Multifocal or unifocal^h^**			0.358			0.45			0.79
Unifocal	75	36		8	103		63	48	
Multifocal	21	6		3	24		17	10	
Stage			0.047			0.035			0.629
Stage I,II	61	34		4	91		53	43	
Stage III,IV	35	8		7	36		27	15	

^a^ MAPK pathway concludes BRAF mutation(for example, BRAF ^V600E^), any fusion genes detected of MAPK pathways and RAS, RET mutation.

^b^ P values derived from Fisher exact test.

^c^ Any fusion genes detected = yes.

^d^ BRAF mutation (for example, BRAF ^V600E^) detected

^e^ Extensive metastasis referred to extensive lymphatic metastasis of neck nodes (for instance, II, III, IV and V region).

^f^ Border observed under ultrasound was classified into clear and obscure ones.

^g^ Calcification observed under ultrasound.

^h^ Multifocal = multi primary foci of papillary thyroid cancer.

## DISCUSSION

In this study, we carried a targeted massively paralleled sequencing assay in a large series of PTC cases. Being cost-efficient, significantly sensitive and accurate, ThyGenCap^TM^ is a promising tool for better understanding thyroid carcinogenesis and clinical genotyping of PTC.

The occurrence of *BRAF* mutations was 58% (80/138) and it ranked as the highest mutational frequency. Most other mutational frequencies were lower than 5%, consistent with the low mutation frequency found in PTC as reported previously [7]. Chromosomal rearrangements and translocations are the common feature ofPTC and contribute to its pathogenesis. We identified both known and novel fusions in 11 (7.8%) of the 138 informative cases. This is a little lower than what was found in a recent multiple platform analysis of 484 cases (15.3%) [7].

In accordance with previous reports, the most common chromosomal rearrangements involve the *RET*protooncogene, as well as *NTRK3*,*NTRK1*, and *BRAF* genes, although with a significantly lower prevalence [14–17]. Since the identification of the first *RET* partner in a chromosomal rearrangement in 1990 [14], at least 25 different 5′-fusion partner genes of *RET* have been described so far, with *CCDC6–RET* (also known for historical reasons as *RET/PTC1*) being the most common [17]. Remarkably, all breakpoints of the already known *RET* rearrangements occurred within intron 11 or occasionally in intron 10 [17, 18]. Of possible interest is the fact that *UEVLD-RET* in this study, unlike the majority of *RET* rearrangements, occurred in intron 2 of *RET*, resulting in longer rearrangements than the most common *RET* rearrangements.

The prevalence of fusion genes in PTC was more frequent in patients who had been diagnosed at a young age and showed a tendency toward a more malignant behavior of PTC [17, 19]. This at least in part explained the fact that younger patients commonly have clinically more advanced disease at presentation with extensive lymph node metastasis and, sometimes, distant metastasis [20, 21]. In our study, we did not find any relationship between *BRAF* mutation and aggressive histopathological or clinical characteristics of PTC. However, results from other laboratories indicated that *BRAF* mutation, as well as the MAPK pathway were associated with worse prognosis [6], although the association of *BRAF* V600E with more aggressive clinicopathologic features and worse outcome has been under debate [22–24]. The discrepancies may be attributed to methodological differences, tumors from different geographical areas, different PTC variants, and tumor heterogeneity [25]. A large multicenter study with all centers using the same methodology should resolve this issue.

Notably, it has been previously reported that *TERT* promoter mutation is infrequent in PTC but has been identified as an indicator of the worst prognosis of PTC [26]. Although we only detected 2 samples harboring this mutation, both cases suffered from lymph node metastasis recurrence in less than 2 years after total thyroidectomy with lymph node dissection. Remarkably, *TERT* and *BRAF* mutations were coexistent in both patients. Seemingly, either *TERT* alone or the synergistic effect of the two mutations affected the clinical outcome. Our observation supported the recent report that the concomitance of *TERT* and *BRAF* mutations was significantly associated with poorer clinicopathological features [27–29]. Nevertheless, the number of patients included in our subgroups was too limited to draw a definitive conclusion.

Another promising application to improve efficacy of therapy is to pursue a mutational-profile-guided targeted therapy for aggressive and metastatic PTC. The knowledge of alterations in the MAPK pathway, such as *RET/PTC* rearrangements and *BRAF* mutations, will provide oportunities for clinical development of novel treatment strategies for this cancer. Among the new therapeutic approaches, the most promising compounds have been the *BRAF* inhibitors (i.e., vemurafenib) [4, 30] and *RET*-kinase inhibitors (i.e., vandetanib) [31]. Clinical trials for additional agents directed against specific genes or mutations are currently underway and are expected to progressively increase the repertoire of available targeted therapies for cancer [32].

In summary, targeted sequencing assay permits the detection of a large panel of genomic mutations, from point mutations to structural rearrangements. The detection is very sensitive even in the context of tumor heterogeneity or sample impurity as they could be overcome by in-depth sequencing. Further, this approach will prove to be valuable as a discovery tool enabling development of new therapeutic agents or providing prognostic information. Therefore, the Thyroid Cancer Panel method is cost-effective and robust enough to be integrated into routine clinical care for PTC.

## MATERIALS AND METHODS

### Patient and clinicopathological data collection

We carried out a retrospective, single-center study including 138 patients with PTC who were treated with total thyroidectomy between 2012 and 2014 at Beijing Cancer Hospital. This study received ethical approval from the Institutional Review Board of the Peking University School of Oncology, China, and the study was carried out in accordance with approved guidelines. Blood samples from each patient were paired with the tumor samples and served as normal controls to discriminate somatic mutations from germline variations. We retrospectively reviewed the clinical records of these patients. Experienced pathologists made histological diagnosis based on specimens stained with hematoxylin and eosin. Demographic and clinicopathological data of these 138 patients with PTC were summarized in Supplementary Table 1.

### Detection of point mutations and gene fusions by ThyGenCap^TM^ panel

A ThyGenCap^TM^ panel was designed to target 244 cancer-related genes and 20 genes often rearranged in thyroid carcinoma. Details of the genes are provided in Supplementary Table 2. Gene sets were selected based on search results of the literature, the COSMIC database, and the TCGA database.

The amplified DNA was captured by GenCap^TM^ Technologyfrom MyGenostics(Beijing, China). For 244 cancer-related genes, the DNA probes were designed to tile along the exon regions of the target genes, as well as the two most common mutations in TERTpromoter, C228T and C250T,upstream of the TERT ATG site. For 20 genes often rearranged in thyroid carcinoma,the DNA probes were covered whole potential rearranged gene, including introns. The capture experiment was conducted according to manufacturer's protocol. The whole-genomic libraries were hybridized with these probes, adsorbed onto the beads via biotin and streptavidin magnetic beads, and the uncaptured DNA fragments were removed by washing. Then the eluted fragments containing the target genes were enriched by 18 cycles of PCR to generate libraries for sequencing. Libraries were quantified and sequenced for paired-end 100bp using the Illumina HiSeq 2000 sequencer (Illumina Inc., San Diego, CA, USA). The Illumina clean reads were mapped to human genome (GRCh37/hg19) using the BWA program. The paired-end read, uniquely mapped with one end to a target gene and the other to another target gene, is identified as a discordant read pair. If a specific position has three or more discordant read pairs, it would be considered as a potential fusion site. The fusion sites were annotated according to human genome (GRCh37/hg19) from the UCSC database.

Only those mutations that had an allele frequency ≥5% were scored as positive for the mutation. All variants identified by next-generation sequencing with mutant allele frequency greater than 10% were validated by Sanger sequencing. For gene rearrangements, the presence of at least 3 high-quality reads spanning the fusion point of the two split genes confirmed by PCR and Sanger sequencing was required to consider the sample positive for the rearrangement.

### Verification and validation of fusion genes by Sanger sequencing

According to the 5′ and 3′ sequences flanking the breakpoints, respectively,we traced its position on chromosome via UCSC genome and then as a sequence to design proper primers. PCR and Sanger sequencing confirmed the spliced sites.

Furthermore, after breakpoints were validated on DNA level, exons were put together by prediction according to exons spanning the breakpoints. RNA was extracted from tissues and followed by RT-PCR. Specific primers for each fusion gene were designed according to exons put together flanking the breakpoints, respectively. RNA sequences spanning two disparate genes and where the coding frame was predicted were to be maintained in the fusion transcript.

### Fusion gene break-apart fluorescence *in situ* hybridization (FISH) assay

FISH was done on formalin-fixed and paraffin-embedded tumor tissues using probes encompassing the genomic region of all potential gene rearrangement loci (Supplementary Figure 1). The probes used in this study were purchased from Jin Lu Biotechnology Co., Ltd, Shaoxing, China), and the probe clones were from C.H.O.R.I., Children´s Hospital, Oakland Research Institute). Labeling with fluorescein using Nick Translation Kit were purchased from Abbott Molecular, Abbott Park, IL, USA). FISH-positive cases were defined as >15% tumor cells showing classic split signals as previously described [13].

### Statistical analysis

Clinicalpathological characteristics associated with mitogen-activated protein kinase (MAPK) pathway alteration, *BRAF* mutations and fusion genes were evaluated using Pearson's chi-square test or Fisher's exact test. Statistical analysis was conducted using SPSS version 13.0 (SPSS, Chicago, IL, USA). All statistical tests were two-sided at the 0.05 significance level.

## SUPPLEMENTARY MATERIALS FIGURES AND TABLES








