# Supplementary material for: Targeted next generation sequencing identifies somatic mutations and gene fusions in papillary thyroid carcinoma
Source: Oncotarget. 2017 Apr 25;8(28):45784–92. doi: 10.18632/oncotarget.17412 (PMC5542227; doi:10.18632/oncotarget.17412)
Supplement: Supplementary file 1 [file oncotarget-08-45784-s001.pdf]

Targeted next generation sequencing identifies somatic mutations and gene fusions in papillary thyroid carcinoma

SUPPLEMENTARY FIGURE

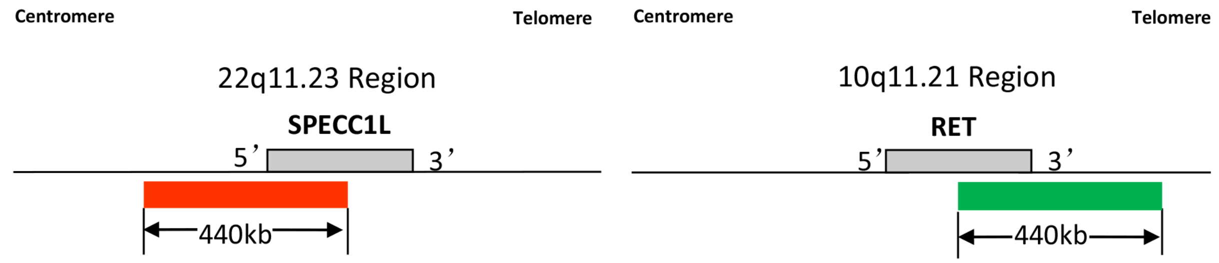

Supplementary Figure 1: Sketch map showed the genes involved rearrangement and probes encompassing the genomic region. The probes used in this study were purchased from Jin Lu Biotechnology Co., Ltd, Shaoxing, China).The BAC clones were from C.H.O.R.I. (Children´s Hospital, Oakland Research Institute) and labeled by nick translation using Nick Translation Kit from Abbott Molecular, Abbott Park, IL, USA). The two differently labeled sets were co-hybridized, resulting in two pairs of adjacent/overlapping red and green FISH signals plus two fused red/green signals (fusion gene) in cells.

1. SPECC1L -RET fusion gene:
SPECC1L-RET Dual Color, Single Fusion Translocation Probe

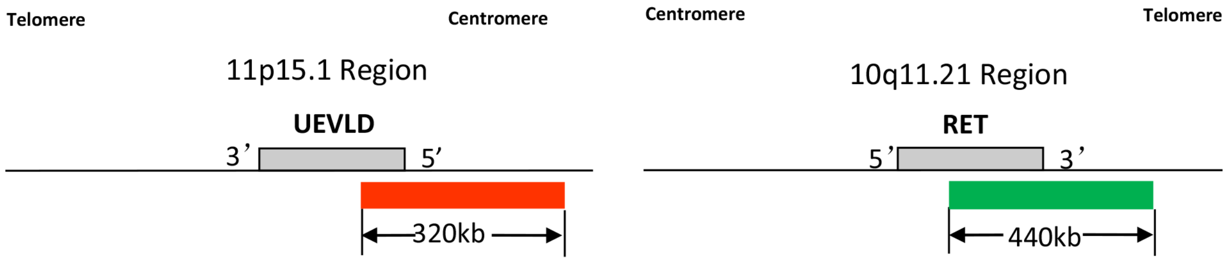

2. UEVLD-RET fusion gene:
UEVLD-RET Dual Color, Single Fusion Translocation Probe

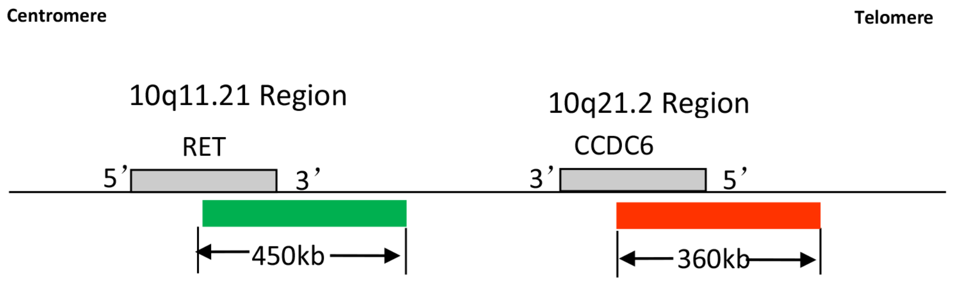

3. CCDC6-RET fusion gene:
CCDC6-RET Dual Color, Single Fusion Translocation Probe

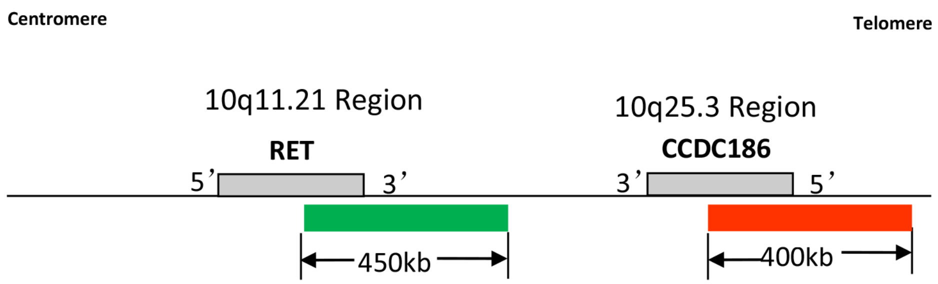

4. CCDC186-RET fusion gene
CCDC186-RET Dual Color, Single Fusion Translocation Probe

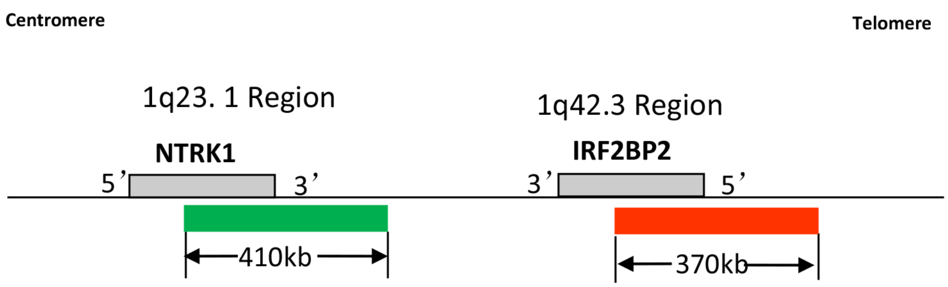

5. IRF2BP2-NTRK1
IRF2BP2-NTRK1 Dual Color, Single Fusion Translocation Probe

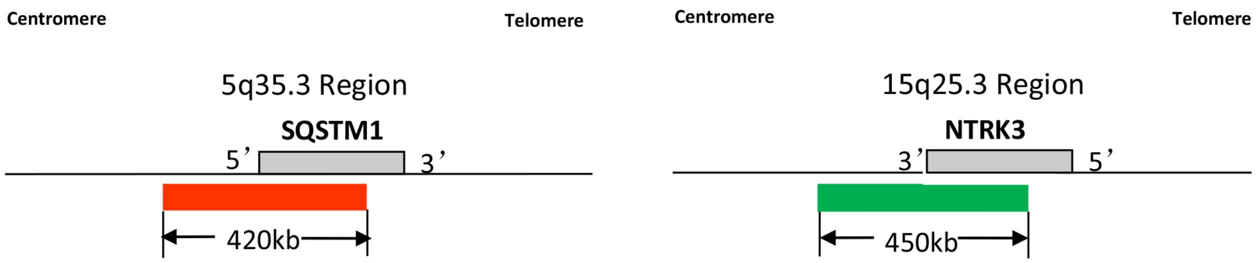

6. SQSTM1-NTRK3 fusion gene:
SQSTM1-NTRK3 Dual Color, Single Fusion Translocation Probe

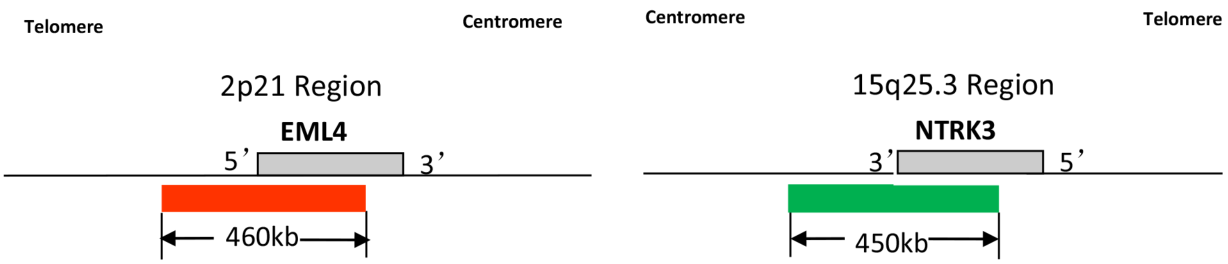

7. EML4-NTRK3 fusion gene:
EML4-NTRK3 Dual Color, Single Fusion Translocation Probe

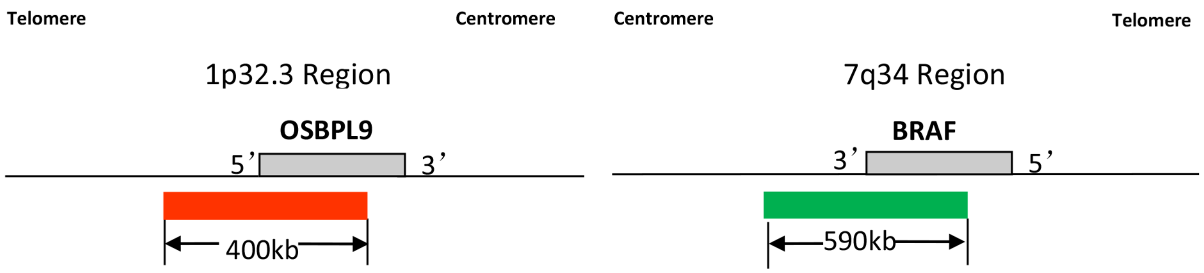

8. OSBPL9-BRAF fusion gene:
OSBPL9-BRAF Dual Color, Single Fusion Translocation Probe

Supplementary Table1: Summarize the clinicopathological features of the 138 patients of PTC

See Supplementary File 1

Supplementary Table 2: Related genes and rearrangement in thyroid carcinoma panel

See Supplementary File 2

Supplementary Table S3: Summary of mutation information

See Supplementary File 3
