# Supplementary material for: Targeted next generation sequencing identifies somatic mutations and gene fusions in papillary thyroid carcinoma
Source: Oncotarget. 2017 Apr 25;8(28):45784–92. doi: 10.18632/oncotarget.17412 (PMC5542227; doi:10.18632/oncotarget.17412)
Supplement: Supplementary file 2 [file oncotarget-08-45784-s002.doc]

**Supplementary Table 1: Summarize the clinicopathological features of the 138 patients of PTC**

| **Preoperative variables** | **No. (%)** |
| --- | --- |
| Gender  Female  Male | 104 (75.4%)  34 (24.6%) |
| Age stratification  ≤30  >30 | 14 (10.1%)  124 (89.9%) |
| Unilateral or bilateral  Unilateral  Bilateral | 127 (92.0%)  11 (8.0%) |
| Tumor size  ≤10mm  10-30mm  >30mm | 55 (39.9%)  69 (50.0%)  14 (10.1%) |
| Border ^a^  Clearance  Obscure | 43 (31.2%)  95 (68.8%) |
| Calcification ^b^  Non-calcification  Calcification | 31 (22.5%)  107 (77.5%) |
| Multifocal or unifocal ^c^  Unifocal  Multifocal | 111 (80.4%)  27 (19.6%) |
| Lymphatic metastasis  Non-metastasis  Metastasis | 74 (53.6%)  64 (46.4%) |
| Extraglandular invasion  Non-invasion  Invation | 90 (65.2%)  48 (34.8%) |
| Extensive metastasis ^d^  Non-extensive  Extensive | 129 (93.5%)  9 (6.5%) |
| Stage  I and II stage  III and IV stage | 95 (68.8%)  43 (31.2%) |
| Histologic variant of papillary thyroid carcinoma |  |
| Classic PTC | 104 (75.4%) |
| Tall cell variant of PTC | 14 (10.1%) |
| Follicular variant of PTC | 11 (8.0%) |
| Diffuse sclerosing variant of PTC | 7 (5.1%) |
| Insular variant of PTC | 2 (1.4%) |

^a^ Border observed under ultrasound was classified into clear and obscure ones.

^b^ Calcification observed under ultrasound = calcification.

^c^ Multifocal = multi primary foci of papillary thyroid cancer.

^d^ Extensive metastasis referred to extensive lymphatic metastasis of neck nodes (for instance, II ,III ,IV and V region).
