# Supplementary material for: Targeted next generation sequencing identifies somatic mutations and gene fusions in papillary thyroid carcinoma
Source: Oncotarget. 2017 Apr 25;8(28):45784–92. doi: 10.18632/oncotarget.17412 (PMC5542227; doi:10.18632/oncotarget.17412)
Supplement: Supplementary file 3 [file oncotarget-08-45784-s003.doc]

**Supplementary Table 2: Related genes and rearrangement in thyroid carcinoma panel**

| **244 cancer related genes, oncogenes and tumor suppressor genes** | | | | | | | | | |
| --- | --- | --- | --- | --- | --- | --- | --- | --- | --- |
| A1CF | ABHD8 | ABL1 | ACVR1B | ADAM12 | ADAM22 | ADPRT | AKAP9 | AKT1 | ALK |
| APC | APOBEC1 | APOBEC2 | APOBEC3A | APOBEC3B | APOBEC3C | APOBEC3D | APOBEC3F | APOBEC3G | APOBEC3H |
| APOBEC4 | ARID1A | ASXL1 | ATM | ATRX | AXIN1 | BAP1 | BCOR | BNC1 | BRAF |
| BRCA1 | BRCA2 | C18orf42 | CAPN3 | CARD11 | CBL | CDC73 | CDH1 | CDKN2A | CDKN2C |
| CEBPA | CHD1 | CHD1L | CHD2 | CHD3 | CHD4 | CHD5 | CHD6 | CHD7 | CHD8 |
| CHD9 | CREBBP | CRLF2 | CSF1R | CTNNA1 | CTNNB1 | CXXC1 | CYB5R1 | CYLD | DAXX |
| DIRC3 | DNAH7 | DNAJB7 | DNMT3A | DPP6 | DSP | EFCAB6 | EGFR | EIF1AX | ELKS |
| EML4 | EP300 | EPHB6 | ERBB2 | ERG | EZH2 | FAM123B | FBXW7 | FCGR3A | FGFR1 |
| FGFR2 | FGFR3 | FLJ36777 | FLT3 | FOXE1 | FOXE1 | FOXL2 | GATA1 | GATA2 | GATA3 |
| GDAP2 | GNA11 | GNAQ | GNAS | GOLGA2 | GOLGA5 | GPN1 | GUSB | H3F3A | HEATR4 |
| HECTD4 | HIST1H3B | HMGA1 | HNF1A | HOOK3 | HRAS | HRPT2 | HSPBAP1 | HSPG2 | HTRA4 |
| IDH1 | IDH2 | IKZF1 | IL7R | IQSEC2 | IRF4 | ITGA10 | JAK1 | JAK2 | JAK3 |
| KCNJ11 | KDR | KIT | KMT2C | KRAS | LUZP2 | MAP2K1 | MAP2K2 | MAP2K4 | MBIP |
| MED12 | MEN1 | MET | MLH1 | MLL2 | MLL3 | MPL | MSH2 | MSH6 | MYD88 |
| MYH2 | NCOA4 | NDUFA13 | NF1 | NF2 | NFE2L2 | NKX2-1 | NOTCH1 | NOTCH2 | NPM1 |
| NRAS | NRG1 | NTRK1 | NTRK3 | OGG1 | P2X7R | PARP3 | PAX5 | PAX8 | PBRM1 |
| PCDHB1 | PCM1 | PDGFRA | PHF6 | PHOX2B | PIK3CA | PIK3R1 | PPARG | PPP1R1B | PPP2R1A |
| PRDM1 | pre-miR146a | PRKAR1A | PSPN | PTCH1 | PTCSC3 | PTEN | PTPN11 | PTPRT | PXK |
| RB1 | RET | RFX6 | RUNX1 | SCN2A | SDHA | SETD2 | SF3B1 | SH2B3 | SH3RF2 |
| SLC20A1 | SLC39A8 | SLC4A3 | SLC9A2 | SMAD4 | SMARCA4 | SMARCB1 | SMO | SOCS1 | SOCS3 |
| SPAG1 | SPN | SRC | SRGAP1 | SRSF2 | SSX5 | STAT3 | STK11 | STXBP5L | SUFU |
| SYTL3 | TAF4B | TDG | TDO2 | TERT | TET2 | TFG | THRAP3 | TNFAIP3 | TNFRSF14 |
| TP53 | TPM3 | TPR | TRIM27 | TRIM33 | TRIP11 | TSC1 | TSC2 | TSHR | TTF-1 |
| U2AF1 | USP24 | VDR | VHL | WDR3 | WT1 | XRCC1 | XRCC2 | XRCC3 | ZC3H7B |
| ZNF331 | ZNF674 | ZNF98 | ZRSR2 |  |  |  |  |  |  |
| **20 chromosomal rearrangement genes** | | | | | | | | | |
| ALK | BCL2 | BCR | BRAF | EGFR | ETV1 | ETV5 | ETV6 | EWSR1 | FGFR2 |
| FTV4 | KLL | NTRK1 | NTRK3 | PDGFRA | PPARG | RARA | RET | ROS1 | TMPRSS2 |
