# Supplementary material for: Targeted next generation sequencing identifies somatic mutations and gene fusions in papillary thyroid carcinoma
Source: Oncotarget. 2017 Apr 25;8(28):45784–92. doi: 10.18632/oncotarget.17412 (PMC5542227; doi:10.18632/oncotarget.17412)
Supplement: Supplementary file 4 [file oncotarget-08-45784-s004.doc]

**Supplementary Table 3: Summary of mutation information**

| Sample_T | Chr | Ref | Alt | Effect | Mutation_type | Gene_symbol | AA_change |
| --- | --- | --- | --- | --- | --- | --- | --- |
| 2014-S03480 | chr12 | G | GA | Frameshift | Insertion | TDG | TDG:NM_003211:exon8:c.965dupA:p.E322fs |
| 2014-S03481 | chr7 | A | T | Nonsynonymous | SNV | BRAF | BRAF:NM_004333:exon15:c.T1799A:p.V600E |
| 2014-S03484 | chr7 | A | T | Nonsynonymous | SNV | BRAF | BRAF:NM_004333:exon15:c.T1799A:p.V600E |
| 2014-S03485 | chr7 | A | T | Nonsynonymous | SNV | BRAF | BRAF:NM_004333:exon15:c.T1799A:p.V600E |
| 2014-S03486 | chr7 | A | T | Nonsynonymous | SNV | BRAF | BRAF:NM_004333:exon15:c.T1799A:p.V600E |
| 2014-S03486 | chr17 | C | T | Nonsynonymous | SNV | TP53 | TP53:NM_001126115:exon3:c.G280A:p.G94S,TP53:NM_001126116:exon3:c.G280A:p.G94S,TP53:NM_001126117:exon3:c.G280A:p.G94S,TP53:NM_001276697:exon3:c.G199A:p.G67S,TP53:NM_001276698:exon3:c.G199A:p.G67S,TP53:NM_001276699:exon3:c.G199A:p.G67S,TP53:NM_001126118:exon6:c.G559A:p.G187S,TP53:NM_000546:exon7:c.G676A:p.G226S,TP53:NM_001126112:exon7:c.G676A:p.G226S,TP53:NM_001126113:exon7:c.G676A:p.G226S,TP53:NM_001126114:exon7:c.G676A:p.G226S,TP53:NM_001276695:exon7:c.G559A:p.G187S,TP53:NM_001276696:exon7:c.G559A:p.G187S,TP53:NM_001276760:exon7:c.G559A:p.G187S,TP53:NM_001276761:exon7:c.G559A:p.G187S |
| 2014-S03487 | chr7 | A | T | Nonsynonymous | SNV | BRAF | BRAF:NM_004333:exon15:c.T1799A:p.V600E |
| 2014-S03489 | chr7 | A | T | Nonsynonymous | SNV | BRAF | BRAF:NM_004333:exon15:c.T1799A:p.V600E |
| 2014-S03489 | chr22 | AATG | A | Frameshift | Deletion | SMARCB1 | SMARCB1:NM_003073:wholegene,SMARCB1:NM_001007468:wholegene |
| 2014-S03492 | chr7 | A | T | Nonsynonymous | SNV | BRAF | BRAF:NM_004333:exon15:c.T1799A:p.V600E |
| 2014-S03493 | chr10 | C | CAAATTTCAAAACACTGGGCAAGACAGTAAATATGCAGACAGGGCCGGGCGTGGTGG | Frameshift | Insertion | NCOA4 | NCOA4:NM_001145262:exon2:c.47_48insAAATTTCAAAACACTGGGCAAGACAGTAAATATGCAGACAGGGCCGGGCGTGGTGG:p.P16fs,NCOA4:NM_001145263:exon2:c.47_48insAAATTTCAAAACACTGGGCAAGACAGTAAATATGCAGACAGGGCCGGGCGTGGTGG:p.P16fs,NCOA4:NM_005437:exon2:c.47_48insAAATTTCAAAACACTGGGCAAGACAGTAAATATGCAGACAGGGCCGGGCGTGGTGG:p.P16fs,NCOA4:NM_001145260:exon3:c.95_96insAAATTTCAAAACACTGGGCAAGACAGTAAATATGCAGACAGGGCCGGGCGTGGTGG:p.P32fs,NCOA4:NM_001145261:exon3:c.95_96insAAATTTCAAAACACTGGGCAAGACAGTAAATATGCAGACAGGGCCGGGCGTGGTGG:p.P32fs |
| 2014-S03493 | chr19 | T | C | Nonsynonymous | SNV | ZNF98 | ZNF98:NM_001098626:exon3:c.A241G:p.T81A |
| 2014-S03494 | chr7 | A | T | Nonsynonymous | SNV | BRAF | BRAF:NM_004333:exon15:c.T1799A:p.V600E |
| 2014-S03497 | chr7 | A | T | Nonsynonymous | SNV | BRAF | BRAF:NM_004333:exon15:c.T1799A:p.V600E |
| 2014-S03498 | chr7 | A | T | Nonsynonymous | SNV | BRAF | BRAF:NM_004333:exon15:c.T1799A:p.V600E |
| 2014-S03499 | chr7 | T | C | Nonsynonymous | SNV | BRAF | BRAF:NM_004333:exon15:c.A1801G:p.K601E |
| 2014-S03500 | chr7 | A | T | Nonsynonymous | SNV | BRAF | BRAF:NM_004333:exon15:c.T1799A:p.V600E |
| 2014-S03501 | chr7 | A | T | Nonsynonymous | SNV | BRAF | BRAF:NM_004333:exon15:c.T1799A:p.V600E |
| 2014-S03502 | chr7 | A | T | Nonsynonymous | SNV | BRAF | BRAF:NM_004333:exon15:c.T1799A:p.V600E |
| 2014-S03503 | chr7 | A | T | Nonsynonymous | SNV | BRAF | BRAF:NM_004333:exon15:c.T1799A:p.V600E |
| 2014-S03503 | chr14 | G | A | Nonsynonymous | SNV | TRIP11 | TRIP11:NM_004239:exon20:c.C5620T:p.P1874S |
| 2014-S03503 | chr1 | C | G | Nonsynonymous | SNV | H3F3A | H3F3A:NM_002107:exon4:c.C344G:p.A115G |
| 2014-S03505 | chr7 | A | T | Nonsynonymous | SNV | BRAF | BRAF:NM_004333:exon15:c.T1799A:p.V600E |
| 2014-S03506 | chr3 | G | A | Nonsynonymous | SNV | RAF1 | RAF1:NM_002880:exon7:c.C776T:p.S259F |
| 2014-S03506 | chr12 | C | A | Nonsynonymous | SNV | KRAS | KRAS:NM_004985:exon2:c.G35T:p.G12V,KRAS:NM_033360:exon2:c.G35T:p.G12V |
| 2014-S03507 | chr7 | A | T | Nonsynonymous | SNV | BRAF | BRAF:NM_004333:exon15:c.T1799A:p.V600E |
| 2014-S03508 | chr7 | A | T | Nonsynonymous | SNV | BRAF | BRAF:NM_004333:exon15:c.T1799A:p.V600E |
| 2014-S03509 | chr7 | A | T | Nonsynonymous | SNV | BRAF | BRAF:NM_004333:exon15:c.T1799A:p.V600E |
| 2014-S03511 | chr1 | G | A | Nonsynonymous | SNV | HSPG2 | HSPG2:NM_001291860:exon57:c.C7441T:p.R2481W,HSPG2:NM_005529:exon57:c.C7438T:p.R2480W |
| 2014-S03513 | chr7 | A | T | Nonsynonymous | SNV | BRAF | BRAF:NM_004333:exon15:c.T1799A:p.V600E |
| 2014-S03514 | chrX | G | A | Nonsynonymous | SNV | CRLF2 | CRLF2:NM_001012288:exon5:c.C335T:p.P112L,CRLF2:NM_022148:exon6:c.C671T:p.P224L |
| 2014-S03515 | chr3 | A | G | Nonsynonymous | SNV | SETD2 | SETD2:NM_014159:exon3:c.T3802C:p.S1268P |
| 2014-S03515 | chr7 | A | T | Nonsynonymous | SNV | BRAF | BRAF:NM_004333:exon15:c.T1799A:p.V600E |
| 2014-S03515 | chr11 | G | C | Nonsynonymous | SNV | ATM | ATM:NM_000051:exon2:c.G56C:p.R19T |
| 2014-S03515 | chr17 | C | G | Nonsynonymous | SNV | NF1 | NF1:NM_000267:exon49:c.C7274G:p.S2425C,NF1:NM_001042492:exon50:c.C7337G:p.S2446C |
| 2014-S03518 | chr1 | C | A | Nonsynonymous | SNV | HSPG2 | HSPG2:NM_001291860:exon49:c.G6266T:p.G2089V,HSPG2:NM_005529:exon49:c.G6263T:p.G2088V |
| 2014-S03520 | chr7 | A | T | Nonsynonymous | SNV | BRAF | BRAF:NM_004333:exon15:c.T1799A:p.V600E |
| 2014-S03521 | chr7 | A | T | Nonsynonymous | SNV | BRAF | BRAF:NM_004333:exon15:c.T1799A:p.V600E |
| 2014-S03521 | chr1 | C | G | Nonsynonymous | SNV | H3F3A | H3F3A:NM_002107:exon4:c.C344G:p.A115G |
| 2014-S03522 | chr7 | A | T | Nonsynonymous | SNV | BRAF | BRAF:NM_004333:exon15:c.T1799A:p.V600E |
| 2014-S03522 | chr11 | GCAC | G | Nonframeshift | Deletion | CBL | CBL:NM_005188:exon1:c.106_108del:p.36_36del |
| 2014-S03523 | chr7 | A | T | Nonsynonymous | SNV | BRAF | BRAF:NM_004333:exon15:c.T1799A:p.V600E |
| 2014-S03523 | chr17 | C | T | Nonsynonymous | SNV | ETV4 | ETV4:NM_001261437:exon2:c.G11A:p.G4D,ETV4:NM_001261438:exon2:c.G11A:p.G4D,ETV4:NM_001079675:exon3:c.G128A:p.G43D,ETV4:NM_001986:exon3:c.G128A:p.G43D |
| 2014-S03523 | chr1 | C | G | Nonsynonymous | SNV | H3F3A | H3F3A:NM_002107:exon4:c.C344G:p.A115G |
| 2014-S03524 | chr7 | A | T | Nonsynonymous | SNV | BRAF | BRAF:NM_004333:exon15:c.T1799A:p.V600E |
| 2014-S03524 | chr19 | GGGC | G | Nonframeshift | Deletion | CEBPA | CEBPA:NM_001285829:exon1:c.207_209del:p.69_70del,CEBPA:NM_001287424:exon1:c.669_671del:p.223_224del,CEBPA:NM_001287435:exon1:c.522_524del:p.174_175del,CEBPA:NM_004364:exon1:c.564_566del:p.188_189del |
| 2014-S03525 | chr7 | G | A | Nonsynonymous | SNV | CARD11 | CARD11:NM_032415:exon16:c.C2081T:p.S694L |
| 2014-S03525 | chr7 | A | T | Nonsynonymous | SNV | BRAF | BRAF:NM_004333:exon15:c.T1799A:p.V600E |
| 2014-S03525 | chr19 | GGGC | G | Nonframeshift | Deletion | CEBPA | CEBPA:NM_001285829:exon1:c.207_209del:p.69_70del,CEBPA:NM_001287424:exon1:c.669_671del:p.223_224del,CEBPA:NM_001287435:exon1:c.522_524del:p.174_175del,CEBPA:NM_004364:exon1:c.564_566del:p.188_189del |
| 2014-S03525 | chrX | T | C | Nonsynonymous | SNV | IQSEC2 | IQSEC2:NM_001111125:exon15:c.A4412G:p.N1471S |
| 2014-S03526 | chr3 | C | A | Nonsynonymous | SNV | BAP1 | BAP1:NM_004656:exon17:c.G2092T:p.V698L |
| 2014-S03526 | chr7 | A | T | Nonsynonymous | SNV | BRAF | BRAF:NM_004333:exon15:c.T1799A:p.V600E |
| 2014-S03528 | chr7 | A | T | Nonsynonymous | SNV | BRAF | BRAF:NM_004333:exon15:c.T1799A:p.V600E |
| 2014-S03528 | chr9 | G | T | Nonsynonymous | SNV | CDKN2A | CDKN2A:NM_000077:exon2:c.C315A:p.D105E,CDKN2A:NM_001195132:exon2:c.C315A:p.D105E,CDKN2A:NM_058195:exon2:c.C358A:p.R120S |
| 2014-S03528 | chr3 | T | G | Nonsynonymous | SNV | ETV5 | ETV5:NM_004454:exon7:c.A536C:p.H179P |
| 2014-S03530 | chr7 | A | T | Nonsynonymous | SNV | BRAF | BRAF:NM_004333:exon15:c.T1799A:p.V600E |
| 2014-S03532 | chr7 | A | T | Nonsynonymous | SNV | BRAF | BRAF:NM_004333:exon15:c.T1799A:p.V600E |
| 2014-S03534 | chr9 | G | T | Nonsynonymous | SNV | TSC1 | TSC1:NM_000368:exon5:c.C256A:p.R86S,TSC1:NM_001162426:exon5:c.C256A:p.R86S |
| 2014-S03534 | chr7 | A | T | Nonsynonymous | SNV | BRAF | BRAF:NM_004333:exon15:c.T1799A:p.V600E |
| 2014-S03536 | chr7 | A | T | Nonsynonymous | SNV | BRAF | BRAF:NM_004333:exon15:c.T1799A:p.V600E |
| 2014-S03536 | chr9 | G | A | Nonsynonymous | SNV | NOTCH1 | NOTCH1:NM_017617:exon23:c.C3835T:p.R1279C |
| 2014-S03536 | chr19 | G | A | Nonsynonymous | SNV | STK11 | STK11:NM_000455:exon9:c.G1168A:p.V390M |
| 2014-S03536 | chr19 | GGGC | G | Nonframeshift | Deletion | CEBPA | CEBPA:NM_001285829:exon1:c.207_209del:p.69_70del,CEBPA:NM_001287424:exon1:c.669_671del:p.223_224del,CEBPA:NM_001287435:exon1:c.522_524del:p.174_175del,CEBPA:NM_004364:exon1:c.564_566del:p.188_189del |
| 2014-S03537 | chr1 | CGCA | C | Nonframeshift | Deletion | ARID1A | ARID1A:NM_006015:exon16:c.3978_3980del:p.1326_1327del,ARID1A:NM_139135:exon16:c.3978_3980del:p.1326_1327del |
| 2014-S03538 | chr2 | CTG | C | Frameshift | Deletion | DNAH7 | DNAH7:NM_018897:exon44:c.8075_8076del:p.T2692fs |
| 2014-S03538 | chr7 | A | T | Nonsynonymous | SNV | BRAF | BRAF:NM_004333:exon15:c.T1799A:p.V600E |
| 2014-S03539 | chr7 | A | T | Nonsynonymous | SNV | BRAF | BRAF:NM_004333:exon15:c.T1799A:p.V600E |
| 2014-S03542 | chr7 | A | T | Nonsynonymous | SNV | BRAF | BRAF:NM_004333:exon15:c.T1799A:p.V600E |
| 2014-S03543 | chr7 | A | T | Nonsynonymous | SNV | BRAF | BRAF:NM_004333:exon15:c.T1799A:p.V600E |
| 2014-S03543 | chr9 | TGTG | T | Nonframeshift | Deletion | NOTCH1 | NOTCH1:NM_017617:exon34:c.7244_7246del:p.2415_2416del |
| 2014-S03543 | chr19 | C | T | Nonsynonymous | SNV | MAP2K2 | MAP2K2:NM_030662:exon11:c.G1195A:p.A399T |
| 2014-S03544 | chr19 | GGGC | G | Nonframeshift | Deletion | CEBPA | CEBPA:NM_001285829:exon1:c.207_209del:p.69_70del,CEBPA:NM_001287424:exon1:c.669_671del:p.223_224del,CEBPA:NM_001287435:exon1:c.522_524del:p.174_175del,CEBPA:NM_004364:exon1:c.564_566del:p.188_189del |
| 2014-S03544 | chrX | C | G | Nonsynonymous | SNV | TFE3 | TFE3:NM_006521:exon1:c.G26C:p.R9P |
| 2014-S03544 | chr3 | T | G | Nonsynonymous | SNV | ETV5 | ETV5:NM_004454:exon7:c.A536C:p.H179P |
| 2014-S03545 | chr12 | C | T | Nonsynonymous | SNV | KRAS | KRAS:NM_004985:exon2:c.G38A:p.G13D,KRAS:NM_033360:exon2:c.G38A:p.G13D |
| 2014-S03546 | chr5 | C | A | Nonsynonymous | SNV | SH3RF2 | SH3RF2:NM_152550:exon7:c.C1204A:p.Q402K |
| 2014-S03546 | chr6 | A | G | Nonsynonymous | SNV | DSP | DSP:NM_001008844:exon2:c.A269G:p.Q90R,DSP:NM_004415:exon2:c.A269G:p.Q90R |
| 2014-S03548 | chr7 | A | T | Nonsynonymous | SNV | BRAF | BRAF:NM_004333:exon15:c.T1799A:p.V600E |
| 2014-S03549 | chr3 | T | G | Nonsynonymous | SNV | ETV5 | ETV5:NM_004454:exon7:c.A536C:p.H179P |
| 2014-S03550 | chr7 | A | T | Nonsynonymous | SNV | BRAF | BRAF:NM_004333:exon15:c.T1799A:p.V600E |
| 2014-S03550 | chr1 | CGCA | C | Nonframeshift | Deletion | ARID1A | ARID1A:NM_006015:exon16:c.3978_3980del:p.1326_1327del,ARID1A:NM_139135:exon16:c.3978_3980del:p.1326_1327del |
| 2014-S03550 | chrX | T | C | Nonsynonymous | SNV | ZNF674 | ZNF674:NM_001039891:exon6:c.A868G:p.I290V,ZNF674:NM_001146291:exon6:c.A850G:p.I284V,ZNF674:NM_001190417:exon6:c.A853G:p.I285V |
| 2014-S03551 | chr7 | A | T | Nonsynonymous | SNV | BRAF | BRAF:NM_004333:exon15:c.T1799A:p.V600E |
| 2014-S03551 | chr9 | C | T | Nonsynonymous | SNV | TTF1 | TTF1:NM_007344:exon2:c.G1267A:p.E423K |
| 2014-S03552 | chr7 | A | T | Nonsynonymous | SNV | BRAF | BRAF:NM_004333:exon15:c.T1799A:p.V600E |
| 2014-S03553 | chr7 | A | T | Nonsynonymous | SNV | BRAF | BRAF:NM_004333:exon15:c.T1799A:p.V600E |
| 2014-S03553 | chr20 | C | CTGACGCCCCAGCCGATCCCGACTCCGGGGCGGCCCG | Nonframeshift | Insertion | GNAS | GNAS:NM_001077490:exon1:c.1189_1190insTGACGCCCCAGCCGATCCCGACTCCGGGGCGGCCCG:p.L397delinsLTPQPIPTPGRPV,GNAS:NM_080425:exon1:c.1376_1377insTGACGCCCCAGCCGATCCCGACTCCGGGGCGGCCCG:p.P459delinsPDAPADPDSGAAR |
| 2014-S03553 | chr20 | C | G | Nonsynonymous | SNV | GNAS | GNAS:NM_001077490:exon1:c.C1189G:p.L397V,GNAS:NM_080425:exon1:c.C1376G:p.P459R |
| 2014-S03554 | chr7 | A | T | Nonsynonymous | SNV | BRAF | BRAF:NM_004333:exon15:c.T1799A:p.V600E |
| 2014-S03556 | chr7 | A | T | Nonsynonymous | SNV | BRAF | BRAF:NM_004333:exon15:c.T1799A:p.V600E |
| 2014-S03558 | chrX | C | G | Nonsynonymous | SNV | TFE3 | TFE3:NM_006521:exon1:c.G26C:p.R9P |
| 2014-S03558 | chrY | G | A | Nonsynonymous | SNV | CRLF2 | CRLF2:NM_001012288:exon5:c.C335T:p.P112L,CRLF2:NM_022148:exon6:c.C671T:p.P224L |
| 2014-S03559 | chr3 | T | C | Nonsynonymous | SNV | CTNNB1 | CTNNB1:NM_001098209:exon3:c.T133C:p.S45P,CTNNB1:NM_001098210:exon3:c.T133C:p.S45P,CTNNB1:NM_001904:exon3:c.T133C:p.S45P |
| 2014-S03559 | chr7 | C | T | Nonsynonymous | SNV | CARD11 | CARD11:NM_032415:exon8:c.G1130A:p.R377Q |
| 2014-S03559 | chr7 | A | T | Nonsynonymous | SNV | BRAF | BRAF:NM_004333:exon15:c.T1799A:p.V600E |
| 2014-S03559 | chr9 | G | A | Nonsynonymous | SNV | NOTCH1 | NOTCH1:NM_017617:exon4:c.C707T:p.T236M |
| 2014-S03559 | chr22 | C | T | Nonsynonymous | SNV | APOBEC3C | APOBEC3C:NM_014508:exon3:c.C406T:p.R136C |
| 2014-S03561 | chr7 | A | T | Nonsynonymous | SNV | BRAF | BRAF:NM_004333:exon15:c.T1799A:p.V600E |
| 2014-S03561 | chr16 | C | T | Nonsynonymous | SNV | AXIN1 | AXIN1:NM_003502:exon9:c.G2218A:p.A740T |
| 2014-S03561 | chrX | C | G | Nonsynonymous | SNV | TFE3 | TFE3:NM_006521:exon1:c.G26C:p.R9P |
| 2014-S03561 | chr1 | C | G | Nonsynonymous | SNV | H3F3A | H3F3A:NM_002107:exon4:c.C344G:p.A115G |
| 2014-S03562 | chr1 | A | G | Nonsynonymous | SNV | NOTCH2 | NOTCH2:NM_001200001:exon3:c.T316C:p.S106P,NOTCH2:NM_024408:exon3:c.T316C:p.S106P |
| 2014-S03562 | chr7 | A | T | Nonsynonymous | SNV | BRAF | BRAF:NM_004333:exon15:c.T1799A:p.V600E |
| 2014-S03562 | chr20 | C | G | Nonsynonymous | SNV | GNAS | GNAS:NM_001077490:exon1:c.C1189G:p.L397V,GNAS:NM_080425:exon1:c.C1376G:p.P459R |
| 2014-S03563 | chr2 | C | A | Nonsynonymous | SNV | MSH2 | MSH2:NM_000251:exon1:c.C14A:p.P5Q |
| 2014-S03563 | chr7 | A | T | Nonsynonymous | SNV | BRAF | BRAF:NM_004333:exon15:c.T1799A:p.V600E |
| 2014-S03564 | chr7 | A | T | Nonsynonymous | SNV | BRAF | BRAF:NM_004333:exon15:c.T1799A:p.V600E |
| 2014-S03564 | chrX | G | A | Nonsynonymous | SNV | CRLF2 | CRLF2:NM_001012288:exon5:c.C335T:p.P112L,CRLF2:NM_022148:exon6:c.C671T:p.P224L |
| 2014-S03567 | chr7 | A | T | Nonsynonymous | SNV | BRAF | BRAF:NM_004333:exon15:c.T1799A:p.V600E |
| 2014-S03567 | chr3 | T | G | Nonsynonymous | SNV | ETV5 | ETV5:NM_004454:exon7:c.A536C:p.H179P |
| 2014-S03570 | chr7 | A | T | Nonsynonymous | SNV | BRAF | BRAF:NM_004333:exon15:c.T1799A:p.V600E |
| 2014-S03571 | chr7 | A | T | Nonsynonymous | SNV | BRAF | BRAF:NM_004333:exon15:c.T1799A:p.V600E |
| 2014-S03572 | chr2 | C | G | Nonsynonymous | SNV | SF3B1 | SF3B1:NM_012433:exon14:c.G1998C:p.K666N |
| 2014-S03572 | chr7 | A | T | Nonsynonymous | SNV | BRAF | BRAF:NM_004333:exon15:c.T1799A:p.V600E |
| 2014-S03573 | chr14 | T | A | Nonsynonymous | SNV | CHD8 | CHD8:NM_001170629:exon37:c.A7635T:p.E2545D,CHD8:NM_020920:exon38:c.A6798T:p.E2266D |
| 2014-S03574 | chr7 | A | T | Nonsynonymous | SNV | BRAF | BRAF:NM_004333:exon15:c.T1799A:p.V600E |
| 2014-S03578 | chr1 | G | A | Nonsynonymous | SNV | TNFRSF14 | TNFRSF14:NM_001297605:exon4:c.G317A:p.R106H,TNFRSF14:NM_003820:exon4:c.G317A:p.R106H |
| 2014-S03579 | chr3 | C | G | Nonsynonymous | SNV | ETV5 | ETV5:NM_004454:exon7:c.G523C:p.A175P |
| 2014-S03579 | chr7 | A | T | Nonsynonymous | SNV | BRAF | BRAF:NM_004333:exon15:c.T1799A:p.V600E |
| 2014-S03580 | chr1 | CGCA | C | Nonframeshift | Deletion | ARID1A | ARID1A:NM_006015:exon16:c.3978_3980del:p.1326_1327del,ARID1A:NM_139135:exon16:c.3978_3980del:p.1326_1327del |
| 2014-S03583 | chr7 | A | T | Nonsynonymous | SNV | BRAF | BRAF:NM_004333:exon15:c.T1799A:p.V600E |
| 2014-S03583 | chr10 | C | G | Nonsynonymous | SNV | ADAM12 | ADAM12:NM_001288973:exon15:c.G1639C:p.E547Q,ADAM12:NM_001288974:exon15:c.G1639C:p.E547Q,ADAM12:NM_001288975:exon15:c.G1639C:p.E547Q,ADAM12:NM_003474:exon15:c.G1648C:p.E550Q,ADAM12:NM_021641:exon15:c.G1648C:p.E550Q |
| 2014-S03585 | chr7 | A | T | Nonsynonymous | SNV | BRAF | BRAF:NM_004333:exon15:c.T1799A:p.V600E |
| 2014-S03585 | chrY | G | A | Nonsynonymous | SNV | CRLF2 | CRLF2:NM_001012288:exon5:c.C335T:p.P112L,CRLF2:NM_022148:exon6:c.C671T:p.P224L |
| 2014-S03587 | chr1 | C | G | Nonsynonymous | SNV | H3F3A | H3F3A:NM_002107:exon4:c.C344G:p.A115G |
| 2014-S03587 | chr7 | A | T | Nonsynonymous | SNV | BRAF | BRAF:NM_004333:exon15:c.T1799A:p.V600E |
| 2014-S03589 | chr6 | A | G | Nonsynonymous | SNV | DSP | DSP:NM_001008844:exon2:c.A269G:p.Q90R,DSP:NM_004415:exon2:c.A269G:p.Q90R |
| 2014-S03589 | chr7 | A | T | Nonsynonymous | SNV | BRAF | BRAF:NM_004333:exon15:c.T1799A:p.V600E |
| 2014-S03590 | chr4 | AGCTGCCGCCGCTGCC | A | Nonframeshift | Deletion | PHOX2B | PHOX2B:NM_003924:exon3:c.765_779del:p.255_260del |
| 2014-S03590 | chr7 | A | T | Nonsynonymous | SNV | BRAF | BRAF:NM_004333:exon15:c.T1799A:p.V600E |
| 2014-S03590 | chr1 | C | G | Nonsynonymous | SNV | H3F3A | H3F3A:NM_002107:exon4:c.C344G:p.A115G |
| 2014-S03591 | chr7 | A | T | Nonsynonymous | SNV | BRAF | BRAF:NM_004333:exon15:c.T1799A:p.V600E |
| 2014-S03591 | chr1 | C | G | Nonsynonymous | SNV | H3F3A | H3F3A:NM_002107:exon4:c.C344G:p.A115G |
| 2014-S03592 | chr7 | A | T | Nonsynonymous | SNV | BRAF | BRAF:NM_004333:exon15:c.T1799A:p.V600E |
| 2014-S03593 | chr7 | A | T | Nonsynonymous | SNV | BRAF | BRAF:NM_004333:exon15:c.T1799A:p.V600E |
| 2014-S03593 | chr17 | GGCA | G | Nonframeshift | Deletion | CHD3 | CHD3:NM_001005271:exon5:c.817_819del:p.273_273del,CHD3:NM_001005273:exon5:c.640_642del:p.214_214del,CHD3:NM_005852:exon5:c.640_642del:p.214_214del |
| 2014-S03593 | chr17 | G | T | Nonsynonymous | SNV | CHD3 | CHD3:NM_001005271:exon6:c.G998T:p.S333I,CHD3:NM_001005273:exon6:c.G821T:p.S274I,CHD3:NM_005852:exon6:c.G821T:p.S274I |
| 2014-S03593 | chr1 | A | G | Nonsynonymous | SNV | ARID1A | ARID1A:NM_006015:exon20:c.A5198G:p.E1733G,ARID1A:NM_139135:exon20:c.A4547G:p.E1516G |
| 2014-S03595 | chr7 | A | T | Nonsynonymous | SNV | BRAF | BRAF:NM_004333:exon15:c.T1799A:p.V600E |
| 2014-S03596 | chr7 | A | T | Nonsynonymous | SNV | BRAF | BRAF:NM_004333:exon15:c.T1799A:p.V600E |
| 2014-S03597 | chr7 | A | T | Nonsynonymous | SNV | BRAF | BRAF:NM_004333:exon15:c.T1799A:p.V600E |
| 2014-S03598 | chr7 | C | T | Nonsynonymous | SNV | SMO | SMO:NM_005631:exon2:c.C517T:p.R173C |
| 2014-S03599 | chr4 | AGCTGCCGCCGCTGCC | A | Nonframeshift | Deletion | PHOX2B | PHOX2B:NM_003924:exon3:c.765_779del:p.255_260del |
| 2014-S03601 | chr7 | A | T | Nonsynonymous | SNV | BRAF | BRAF:NM_004333:exon15:c.1799T>A:p.V600E |
| 2014-S03606 | chr7 | A | T | Nonsynonymous | SNV | BRAF | BRAF:NM_004333:exon15:c.1799T>A:p.V600E |
| 2014-S03617 | chr7 | A | T | Nonsynonymous | SNV | BRAF | BRAF:NM_004333:exon15:c.1799T>A:p.V600E |
| 2014-S03620 | chr7 | A | T | Nonsynonymous | SNV | BRAF | BRAF:NM_004333:exon15:c.1799T>A:p.V600E |
| 2014-S03631 | chr7 | A | T | Nonsynonymous | SNV | BRAF | BRAF:NM_004333:exon15:c.1799T>A:p.V600E |
| 2014-S03634 | chr7 | A | T | Nonsynonymous | SNV | BRAF | BRAF:NM_004333:exon15:c.1799T>A:p.V600E |
| 2014-S03645 | chr7 | A | T | Nonsynonymous | SNV | BRAF | BRAF:NM_004333:exon15:c.1799T>A:p.V600E |
| 2014-S03649 | chr7 | A | T | Nonsynonymous | SNV | BRAF | BRAF:NM_004333:exon15:c.1799T>A:p.V600E |
| 2014-S03653 | chr1 | C | T | Nonsynonymous | SNV | NOTCH2 | NOTCH2:NM_001200001:exon1:c.61G>A:p.A21T,NOTCH2:NM_024408:exon1:c.61G>A:p.A21T |
| 2014-S03653 | chr7 | A | T | Nonsynonymous | SNV | BRAF | BRAF:NM_004333:exon15:c.1799T>A:p.V600E |
| 2014-S03653 | chr1 | CGG | C | Frameshift | Deletion | NOTCH2 | NOTCH2:NM_001200001:exon1:c.17_18del:p.P6fs,NOTCH2:NM_024408:exon1:c.17_18del:p.P6fs |
| 2014-S03657 | chr7 | A | T | Nonsynonymous | SNV | BRAF | BRAF:NM_004333:exon15:c.1799T>A:p.V600E |
| 2014-S03660 | chr7 | A | T | Nonsynonymous | SNV | BRAF | BRAF:NM_004333:exon15:c.1799T>A:p.V600E |
| 2014-S03663 | chr7 | A | T | Nonsynonymous | SNV | BRAF | BRAF:NM_004333:exon15:c.1799T>A:p.V600E |
| 2014-S03666 | chr7 | A | T | Nonsynonymous | SNV | BRAF | BRAF:NM_004333:exon15:c.1799T>A:p.V600E |
| 2014-S03666 | chr9 | G | A | Nonsynonymous | SNV | PAX5 | PAX5:NM_001280550:exon7:c.791C>T:p.T264I,PAX5:NM_001280549:exon8:c.878C>T:p.T293I |
| 2014-S03670 | chr5 | T | C | Nonsynonymous | SNV | CSF1R | CSF1R:NM_001288705:exon7:c.1085A>G:p.H362R,CSF1R:NM_005211:exon8:c.1085A>G:p.H362R |
| 2014-S03617 | chr22 | A | G | Nonsynonymous | SNV | APOBEC3A | APOBEC3A_B:NM_001193289:exon4:c.475A>G:p.K159E,APOBEC3A:NM_001270406:exon4:c.421A>G:p.K141E,APOBEC3A:NM_145699:exon4:c.475A>G:p.K159E |
| 2014-S03493 | chr5 | C | T | Nonsynonymous | SNV | SDHA | SDHA:NM_001294332:exon9:c.C1202T:p.A401V,SDHA:NM_004168:exon10:c.C1346T:p.A449V |
| 2014-S03499 | chr7 | CT | C | Frameshift | Deletion | KMT2C | KMT2C:NM_170606:exon38:c.8390delA:p.K2797fs |
| 2014-S03499 | chr9 | G | T | Stopgain | SNV | GNAQ | GNAQ:NM_002072:exon2:c.C303A:p.Y101X |
| 2014-S03617 | chr22 | C | T | Nonsynonymous | SNV | APOBEC3A | APOBEC3A_B:NM_001193289:exon4:c.478C>T:p.H160Y,APOBEC3A:NM_001270406:exon4:c.424C>T:p.H142Y,APOBEC3A:NM_145699:exon4:c.478C>T:p.H160Y |
| 2014-S03653 | chr14 | T | G | Nonsynonymous | SNV | CHD8 | CHD8:NM_001170629:exon37:c.7499A>C:p.H2500P,CHD8:NM_020920:exon38:c.6662A>C:p.H2221P |
| 2014-S03609 | chr11 | T | C | Nonsynonymous | SNV | HRAS | HRAS:NM_005343:exon3:c.182A>G:p.Q61R,HRAS:NM_176795:exon3:c.182A>G:p.Q61R,HRAS:NM_001130442:exon3:c.182A>G:p.Q61R |
| 3480 | chr1 | C | T | Nonsynonymous | SNV | ARID1A | ARID1A:NM_006015:exon18:c.4856C>T:p.P1619L,ARID1A:NM_139135:exon18:c.4205C>T:p.P1402L |
| 3480 | chr11 | A | T | Nonsynonymous | SNV | ATM | ATM:NM_000051:exon27:c.4078A>T:p.S1360C |
| 3525 | chr1 | - | CGG | Nonframeshift | Insertion | ARID1A | ARID1A:NM_006015:exon1:c.362_363insCGG:p.P121delinsPG,ARID1A:NM_139135:exon1:c.362_363insCGG:p.P121delinsPG |
| 3510 | chr11 | C | T | Nonsynonymous | SNV | ATM | ATM:NM_000051:exon30:c.4549C>T:p.L1517F |
| 3532 | chrX | T | C | Nonsynonymous | SNV | EIF1AX | EIF1AX:NM_001412:exon6:c.421A>G:p.I141V |
| 3540 | chr17 | C | T | Nonsynonymous | SNV | TP53 | TP53:NM_001276760:exon8:c.719G>A:p.G240E,TP53:NM_001126115:exon4:c.440G>A:p.G147E,TP53:NM_001126118:exon7:c.719G>A:p.G240E,TP53:NM_001276699:exon4:c.359G>A:p.G120E,TP53:NM_001276761:exon8:c.719G>A:p.G240E,TP53:NM_001126114:exon8:c.836G>A:p.G279E,TP53:NM_001126113:exon8:c.836G>A:p.G279E,TP53:NM_001276698:exon4:c.359G>A:p.G120E:TP53:NM_000546:exon8:c.836G>A:p.G279E,TP53:NM_001126116:exon4:c.440G>A:p.G147E,TP53:NM_001276696:exon8:c.719G>A:p.G240E:TP53:NM_001276697:exon4:c.359G>A:p.G120E,TP53:NM_001126117:exon4:c.440G>A:p.G147E,TP53:NM_001276695:exon8:c.719G>A:p.G240E,TP53:NM_001126112:exon8:c.836G>A:p.G279E |
| 3557 | chr8 | A | G | Nonsynonymous | SNV | MYC | MYC:NM_002467:exon2:c.581A>G:p.Q194R |
| 3561 | chr17 | T | A | Nonsynonymous | SNV | TP53 | TP53:NM_001276760:exon10:c.956A>T:p.E319V,TP53:NM_001126115:exon6:c.677A>T:p.E226V,TP53:NM_001126118:exon9:c.956A>T:p.E319V,TP53:NM_001276761:exon10:c.956A>T:p.E319V,TP53:NM_000546:exon10:c.1073A>T:p.E358V,TP53:NM_001276697:exon6:c.596A>T:p.E199V,TP53:NM_001126112:exon10:c.1073A>T:p.E358V |
| 3596 | chr2 | G | A | Nonsynonymous | SNV | ALK | ALK:NM_004304:exon11:c.2009C>T:p.S670L |
| 3632 | chr11 | C | A | Stopgain | SNV | ATM | ATM:NM_000051:exon38:c.5697C>A:p.C1899X |
| 3633 | chr11 | C | A | Stopgain | SNV | ATM | ATM:NM_000051:exon38:c.5697C>A:p.C1900X |
| 3643 | chr10 | G | C | Nonsynonymous | SNV | PTEN | PTEN:NM_000314:exon5:c.457G>C:p.D153H |
| 2014-S03653-T | chr1-120611960-120611960 | C | T | Nonsynonymous | SNV | NOTCH2 | NM_024408 exon6 c.953G>T p.R318L NM_001200001 exon6 c.953G>T p.R318L NOTCH2 NM_024408 exon6 c.953G>T p.R318L |
| 2014-S03653-T | chr1-120612002-120612004 | CGG | C | Frameshift | Deletion | NOTCH2 | NM_001200001:exon1:c.17_18del:p.P6fs,NOTCH2:NM_024408:exon1:c.17_18del:p.P6fs |
| 2014-S03660-T | chr1-161518214-161518214 | T | C | Nonsynonymous | SNV | FCGR3A | NM_000569:exon3:c.424A>G:p.I142V,FCGR3A:NM_001127592:exon3:c.421A>G:p.I141V,FCGR3A:NM_001127593:exon4:c.316A>G:p.I106V,FCGR3A:NM_001127595:exon4:c.316A>G:p.I106V,FCGR3A:NM_001127596:exon4:c.313A>G:p.I105V |
| 2014-S03660-T | chr1-161518286-161518286 | C | T | Nonsynonymous | SNV | FCGR3A | NM_000569::exon3:c.352G>A:p.D118N,FCGR3A:NM_001127592:exon3:c.349G>A:p.D117N,FCGR3A:NM_001127593:exon4:c.244G>A:p.D82N,FCGR3A:NM_001127595:exon4:c.244G>A:p.D82N,FCGR3A:NM_001127596:exon4:c.241G>A:p.D81N |
| 2014-S03666-T | chr9-36840623-36840623 | G | A | Nonsynonymous | SNV | PAX5 | NM_001280550:exon7:c.791C>T:p.T264I,PAX5:NM_001280549:exon8:c.878C>T:p.T293I |
| 2014-S03670-T | chr5-149450132-149450132 | T | C | Nonsynonymous | SNV | CSF1R | NM_001288705:exon7:c.1085A>G:p.H362R,CSF1R:NM_005211:exon8:c.1085A>G:p.H362R |
| 2014-S03617-T | chr22-39358108-39358108 | A | G | Nonsynonymous | SNV | APOBEC3A,APOBEC3A_B NM_001193289:exon4:c.475A>G:p.K159E,APOBEC3A:NM_001270406:exon4:c.421A>G:p.K141E,APOBEC3A:NM_145699:exon4:c.475A>G:p.K159E | |
| 2014-S03617-T | chr22-39358111-39358111 | C | T | Nonsynonymous | SNV | APOBEC3A,APOBEC3A_B NM_001193289:exon4:c.478C>T:p.H160Y,APOBEC3A:NM_001270406:exon4:c.424C>T:p.H142Y,APOBEC3A:NM_145699:exon4:c.478C>T:p.H160Y | |
| 2014-S03653-T | chr3-178937755-178937755 | T | C | Nonsynonymous | SNV | PIK3CA | NM_006218:exon13:c.1930T>C:p.Y644H |
| 2014-S03653-T | chr14-21854019-21854019 | T | G | Nonsynonymous | SNV | CHD8 | NM_001170629:exon37:c.7499A>C:p.H2500P,CHD8:NM_020920:exon38:c.6662A>C:p.H2221P |
| 2014-S03582 | chr14 | A | C | Nonsynonymous | SNV | TSHR | TSHR:NM_000369:exon10:c.1090A>C:p.I364L |
| 2014-S03553 | chr14 | C | T | splicing | SNV | TSHR | TSHR:NM_001142626:exon9:c.693-4C>T |
| 2014-S03532 | chrX | T | C | Nonsynonymous | SNV | EIF1AX | EIF1AX:NM_001412:exon6:c.421A>G:p.I141V |
| 2014-S03520 | chr5 | C | T | Nonsynonymous | SNV | TERT | TERT:NM_001193376:exon4:c.1949G>A:p.R650K,TERT:NM_198253:exon4:c.1949G>A:p.R650K |
| 2014-S03523 | chr17 | C | T | Nonsynonymous | SNV | ETV4 | ETV4:NM_001261437:exon2:c.11G>A:p.G4D:ETV4:NM_001986:exon3:c.128G>A:p.G43D,ETV4:NM_001261438:exon2:c.11G>A:p.G4D,ETV4:NM_001079675:exon3:c.128G>A:p.G43D |
| 2014-S03586 | chr3 | T | C | Nonsynonymous | SNV | ETV5 | ETV5:NM_004454:exon8:c.800A>G:p.H267R |
| 2014-S03620 | chr17 | A | G | Nonsynonymous | SNV | ETV4 | ETV4:NM_001261437:exon9:c.833T>C:p.F278S,ETV4:NM_001986:exon10:c.950T>C:p.F317S:ETV4:NM_001261438:exon9:c.833T>C:p.F278S:ETV4:NM_001261439:exon2:c.119T>C:p.F40S,ETV4:NM_001079675:exon10:c.950T>C:p.F317S |
| 2014-S03621 | chr17 | A | G | Nonsynonymous | SNV | ETV4 | ETV4:NM_001261437:exon9:c.833T>C:p.F278S,ETV4:NM_001986:exon10:c.950T>C:p.F317S:ETV4:NM_001261438:exon9:c.833T>C:p.F278S:ETV4:NM_001261439:exon2:c.119T>C:p.F40S,ETV4:NM_001079675:exon10:c.950T>C:p.F317S |
| 2014-S03622 | chr17 | A | G | Nonsynonymous | SNV | ETV4 | ETV4:NM_001261437:exon9:c.833T>C:p.F278S,ETV4:NM_001986:exon10:c.950T>C:p.F317S:ETV4:NM_001261438:exon9:c.833T>C:p.F278S:ETV4:NM_001261439:exon2:c.119T>C:p.F40S,ETV4:NM_001079675:exon10:c.950T>C:p.F317S |
| 2014-S03547 | chr5 | C | T | Nonsynonymous | SNV | TERT | TERT:NM_001193376:exon4:c.1949G>A:p.R650K,TERT:NM_198253:exon4:c.1949G>A:p.R650K |
| 2014-S03510 | chr3 | AAAGTCA | - | Nonsynonymous | SNV | splicing | deletion：PIK3CA |
| 2014-S03541 | chr3 | A | G | Nonsynonymous | SNV | PIK3CA | PIK3CA:NM_006218:exon7:c.1173A>G: |
| 2015-S03518 | chr10 | G | A | Nonsynonymous | SNV | RET | RET:NM_020630:exon3:c.341G>A:p.R114H,RET:NM_020975:exon3:c.341G>A:p.R114H |
